# Lessons from an eight-country community health data harmonization collaborative

**DOI:** 10.1080/16549716.2021.2015743

**Published:** 2022-02-04

**Authors:** Madeleine Ballard, Helen Elizabeth Olsen, Caroline Whidden, Daniele Ressler, Lynn Metz, Anoushka Millear, Daniel Palazuelos, Nandini Choudhury, Fabien Munyaneza, Rene Diane, Kelly Lue, P. Émile Bobozi, Anant Raut, Andriamanolohaja Ramarson, Mamy Andrianomenjanahary, Karen Finnegan, Carey Westgate, Wycliffe Omwanda, Leping Wang, David Citrin, Ash Rogers, Moses Banda Aron, Molly Christiansen, Agnes Watsemba, Rehan Adamjee, Amanda Yembrick

**Affiliations:** a Community Health Impact Coalition, Icahn School of Medicine at Mount Sinai, New York, NY, USA; b Medic, San Francisco, CA, USA; c Muso, Bamako, Mali; d Lwala Community Alliance, Rongo, Kenya; e Thinkmd, Burlington, VT, USA; f Partners in Health, Brigham and Women’s Hospital, Harvard Medical School, Boston, MA, USA; g Icahn School of Medicine at Mount Sinai; Possible, New York, NY, USA; h Partners in Health, Neno, Malawi; i One to One Children’s Fund, Cape Town, South Africa; j Integrate Health/Santé Intégrée, Lomé, Togo; k Integrate Health/Santé Intégrée, Kara, Togo; l PIVOT, Ranomafana, Madagascar; m Harvard Medical School, PIVOT, Boston, MA, USA; n Community Health Impact Coalition, New York, NY, USA; o Department of Sociology, Boston University Graduate School of Arts & Sciences, Boston, MA, USA; p Possible; Medic; University of Washington; Icahn School of Medicine at Mount Sinai, Seattle, WA, USA; q Lwala Community Alliance, Nashville, TN, USA; r Living Goods, San Francisco, CA, USA; s Living Goods, Kampala, Uganda; t VITAL Pakistan, Karachi, Pakistan

**Keywords:** Community health workers, COVID-19

## Abstract

Background: Community health workers (CHWs) are individuals who are trained and equipped to provide essential health services to their neighbors and have increased access to healthcare in communities worldwide for more than a century. However, the World Health Organization (WHO) Guideline on Health Policy and System Support to Optimize Community Health Worker Programmes reveals important gaps in the evidentiary certainty about which health system design practices lead to quality care. Routine data collection across countries represents an important, yet often untapped, opportunity for exploratory data analysis and comparative implementation science. However, epidemiological indicators must be harmonized and data pooled to better leverage and learn from routine data collection.Methods: This article describes a data harmonization and pooling Collaborative led by the organizations of the Community Health Impact Coalition, a network of health practitioners delivering community-based healthcare in dozens of countries across four WHO regions.Objectives: The goals of the Collaborative project are to; (i) enable new opportunities for cross-site learning; (ii) use positive and negative outlier analysis to identify, test, and (if helpful) propagate design practices that lead to quality care; and (iii) create a multi-country ‘brain trust’ to reinforce data and health information systems across sites.Results: This article outlines the rationale and methods used to establish a data harmonization and pooling Collaborative, early findings, lessons learned, and directions for future research.

## Background

Community health workers (CHWs) are individuals who are trained and equipped to provide essential health services to their neighbors and have increased access to healthcare in communities worldwide for more than a century [1]. Rigorous research indicates that CHWs can safely deliver promotive, preventative, diagnostic, and treatment services as diverse as administering injectable contraceptives to providing one-on-one psychosocial support to reduce maternal depression [2]. Ultimately, the work of CHWs can reduce child morbidity and mortality while providing considerable return on investment; modeling suggests that every one USD invested in CHW programs can yield a return of up to ten USD through both saved lives and job creation [3,4].

The 2018 World Health Organization (WHO) *Guideline on Health Policy and System Support to Optimize Community Health Worker Programmes*, however, revealed important gaps in the evidentiary certainty about which health system design practices lead to quality care [5]. In response, members of the Community Health Impact Coalition (‘the Coalition’), a network of health practitioners working to make professionalized community health workers a norm worldwide, set up a data harmonization collaborative (‘the Collaborative’) to pool CHW program data, jointly engage in exploratory data analysis, and generate implementation insights to help close critical evidence gaps.

The goals of the data harmonization and pooling project are to; (i) enable new opportunities for cross-site learning; (ii) use positive and negative outlier analysis to identify potential quality improvement practices for testing and, if helpful, propagation; and (iii) create a multi-country ‘brain trust,’ a space to exchange knowledge and experiences, to reinforce data and health information systems across various sites, and, ultimately, to contribute to an aggregate view of what can be achieved through high-impact community health delivery worldwide.

The implementation sites of the Coalition organizations, which cover more than 40 countries and four WHO regions, represent an important opportunity for collaborative data-sharing, exploratory data analysis, and comparative implementation science. To better leverage and learn from routine data collection, however, site-specific indicators that assess program performance must be harmonized and data pooled. This article outlines; (i) the rationale and methods used to establish data harmonization and pooling Collaborative, (ii) early findings, (iii) lessons learned, and (iv) directions for future research.

## Planning the collaborative

### Indicator selection

From February to June 2019, the service delivery indicators measured by eleven Coalition organizations were collated and grouped according to type. Nominal group technique was used in the context of three focus groups involving the research, monitoring, and evaluation teams, and leadership of Coalition organizations to establish a set of indicators with which to begin the harmonization and pooling [6].

The group achieved consensus on a set of nine service delivery indicators that measured the speed, coverage, and quality of CHW care. The intention was to choose a list of indicators that included both aspirational measures and indicators reflective of what organizations were already monitoring (Table 1). The aspiration was to make a statement about what community health programs ought to strive to measure and monitor, based on members’ collective experience in CHW programming. For instance, more organizations measured the proportion of children assessed within 72 hours of symptom onset rather than within 24 hours; however, as malaria and other childhood illnesses can often cause suffering and death within the first 24 hours, an ambitious target was set (metric 1, Table 1) [7]. Likewise, rather than simply focusing on metrics pertaining to service coverage, a deliberate effort was made to triangulate indicators for quality and speed of care.

**Table 1.** Initial indicators chosen for community health data harmonization and pooling

		Metric	Description	Numerator definition	Numerator source	Denominator definition	Denominator source
S P E E D	1	Integrated community case manage- ment (iCCM) Speed	Percentage of children assessed, with a symptom of malaria, diarrhea, or pneumonia, within 24 hours of symptom onset	Number of children assessed, with a symptom of malaria, diarrhea, or pneumonia, within 24 hours of symptom onset	CHW activity data	Number of children assessed with a symptom of malaria, diarrhea, or pneumonia	CHW activity data
C O V E R A G E	2	Pregnancy Speed	Percentage of pregnancies registered in first trimester	Number of pregnancies registered in first trimester	CHW activity data	Number of new pregnancies registered in the month	CHW activity data
	3	Postnatal Care (PNC) Speed	Percentage of women with home delivery receiving 1st PNC visit within 48 hours of delivery	Number of women with home delivery who received 1st PNC visit within 48 hours of delivery this month	CHW activity data	Number of women giving birth at home this month	CHW activity data
	4	Proactive Coverage	Percentage of households visited at least once per month (where family was home)	Number of households visited once or more per month	CHW activity data	Number of households in CHW catchment area	CHW activity data or population survey
	5	U5 Coverage	Number of assessments of children under 5 years of age	Number of assessments of children under 5	CHW activity data	-	-
	6	Contraceptive Coverage	Contraceptive prevalence rate	Number of women 15–49 using modern family planning	Facility data + CHW activity data	Number of women 15–49 years old	CHW activity data
	7	Deliveries Coverage	Percentage of deliveries at a health facility	Number of women giving birth in a health institution under the care and supervision of trained healthcare providers	Facility data or CHW activity data	Number of women giving birth	Facility data + CHW activity data
Q U A L I T Y	8	Treatment Quality	Percentage of correct pre-referral treatment administered by CHW, when recommended	Number of people who received the correct treatment at their doorstep (before being referred, if referral is recommended)	CHW activity data	Total number of consultations	CHW activity data
	9	Referrals Quality	Percentage of referral follow-ups with health facility visit confirmed	Number of referral follow ups with health facility visit confirmed	CHW activity data	Number of referral follow ups	CHW activity data

Additional pragmatic considerations included; (i) selecting metrics representing different health areas (e.g. child health, maternal health, all referral types, etc.) and (ii) recognizing that non-governmental organizations, such as those that make up the Coalition, ought to be aligning indicators and systems of measurement to existing public sector healthcare systems [8]. The second consideration led us to select indicators already in broad use (e.g. percentage of deliveries at a health facility).

Service delivery metrics were chosen for two reasons. First, while there is consensus on ‘impact’ indicators for many of the services provided by CHWs (e.g. under-five mortality, maternal mortality), there is less global consensus on what to measure on a month-to-month or quarterly basis to ensure that health delivery is on track to achieve such impact, making the data pooling required for cross-site synthesis and learning often impossible [9]. While this has since improved with the 2021 release of the *Guidance for Community Health Worker Strategic Information and Service Monitoring* and CHW-led work on construct definition, it is still necessary to identify which service delivery metrics best predict impact outcomes and to drive uptake of harmonized definitions [10,11]. Second, in a context in which hundreds of randomized trials demonstrate the efficacy of CHW programs [3,12], large-scale programs often produce no results, and the capture and analysis of service delivery implementation data on program speed, quality, and coverage is needed to foster necessary quality improvement [13].

### Priority-setting

To determine the logistics and potential use cases for data harmonization and pooling, the Coalition undertook a series of one-on-one calls with representatives from the monitoring, evaluation, learning, and/or research teams at eleven of the Coalition organizations. The first took place in August and September 2019 following the selection of potential indicators, but before data had been shared. The conversations allowed for a mapping of each organization’s current data infrastructure, planned improvements, existing data use, extent of historical data, and barriers to participation (see Appendix I for a selection of summary charts). These initial discussions also captured each organization’s aspirations and ideas for the project.

The second set of one-on-one calls took place in October 2019 after the first round of data sharing by those who had committed to participate (see discussion of data-sharing infrastructure in the next section). These conversations were structured around identifying and overcoming barriers to participation, exploring initial discrepancies in data definitions, selecting priority use cases, and determining scheduling preferences.

Participating organizations were primarily interested in observing how their performance compared to that of others and testing strategies to improve both data quality and health outcomes. The possibility of new, joint, prospective, multi-country studies based on insights from the aggregated and pooled data was likewise attractive. Ultimately, three goals emerged: (i) enable new opportunities for cross-site learning; (ii) use positive and negative outlier analysis to identify potential quality improvement practices to test and propagate; and (iii) create a multi-country ‘brain trust’ for data system strengthening.

### Data-sharing infrastructure

Prior to pooling data, Coalition organizations co-drafted and signed data-sharing agreements (Appendix II) to provide a framework for the project and protect shared data. All civil society organizations received permission from the public health system in which they worked before entering the agreements. On the basis of these agreements, the Coalition set up a Health Insurance Portability and Accountability Act (HIPAA)–compliant data drop and storage system using OwnCube with a quarterly data-sharing cadence for participating partners [14].

### Meeting and analysis cadence

On the basis of one-on-one conversations, the Coalition met on a quarterly basis beginning in January 2020. Quarterly calls were initially designed to consist of a (i) quality improvement session and (ii) a data system question discussion:

(i) Quality improvement session (60 minutes): examination of anonymized plots; presentations by high performers, big decliners, and/or big improvers who consent to de-anonymize themselves; questions; discussion about interpretation; and hypothesis generation (e.g., examination of pregnancy speed and delivery coverage data series, including outlier results)

(ii) Data systems question discussion (30 minutes): Open discussion on a data systems question raised by the group (e.g., How does your organization perform data quality checks?)

In early 2021, the Coalition decided to switch from 90-minute quarterly calls to 60-minute bi-monthly calls that alternated between quality improvement and data systems work. More frequent touch points were thought to be better for improving group cohesion and for the speed of analysis generation.

## Indicator alignment

Data submission and pooling have been conducted quarterly since early 2020. While the initial indicator alignment was nonexistent, data harmonization has improved over time.

(1) At baseline, organizations had no indicators in common.

Before the nine indicators of focus were chosen, all 800+ monthly metrics used by coalition organizations were pooled and grouped according to type (Appendix III and IV). Despite similar community health service delivery models, no one single monthly indicator was common to all eleven initial organizations at the start of the collaboration. The most frequently tracked indicator was the number or percentage of household visits in the previous month, which was tracked by just over half of the organizations (Table 2).

**Table 2.** Geographic scope of the collaborative today

Organization	Country	Number of Districts/Counties	Number of CHWs	Number of indicators shared
Integrate Health	Togo	4	139	7
Muso	Mali	1	225	9
Lwala	Kenya	1	402	8
Partners In Health – Malawi	Malawi	1	1,128	5
Living Goods	Uganda	19	4,511	7
Living Goods	Kenya	5	1,656	8
Wuqu’ Kawoq	Guatemala	10	50	3
Possible	Nepal	2	104	4
VITAL	Pakistan	6	100	4

Given the strategic and programmatic alignment of the Coalition members, this was a surprising finding that may reflect the influence of operating context, funder reporting requirements, and organizational capacity.

(2) Most organizations initially tracked coverage indicators, not quality indicators.

**Figure 1.**
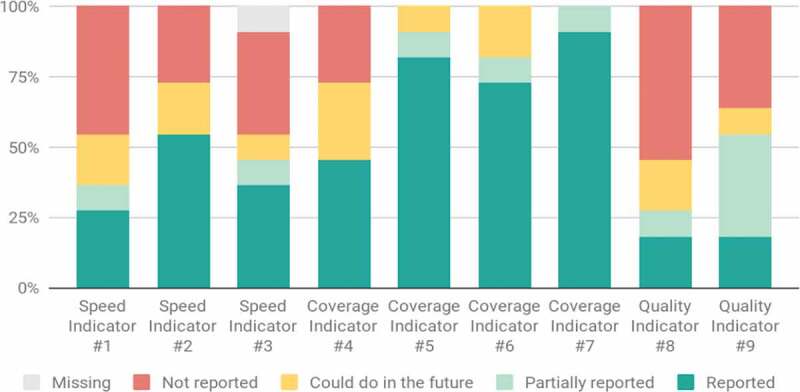
Initial reporting levels across the nine indicators.

In the third and fourth quarters of 2019, Coalitions pooled historical and monthly data for each of the nine initial indicators. While most Coalition members were able to provide coverage data, few were able to provide data on quality indicators (Figure 1).

The three most commonly reported indicators were coverage indicators: U5 assessment coverage, delivery coverage, and contraceptive coverage. The next most reported metrics are those focused on speed, specifically, pregnancy speed and PNC speed. While iCCM speed was initially only reported by three of the Coalition partners, this was still higher than the reporting rates for the two quality indicators.

(3) Definitional alignment and reporting are currently at nearly 100%.

**Figure 2.**
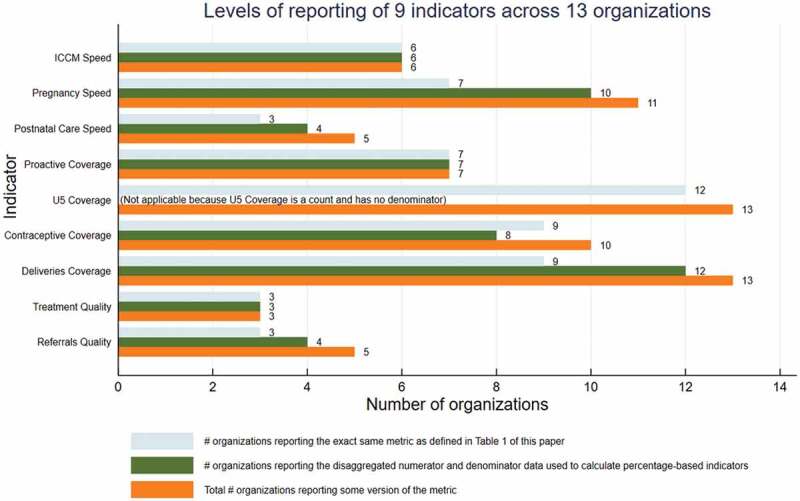
Current status of indicators reporting.

The majority of the current organizations report most of the indicators, using identical definitions for numerators and denominators (Figure 2).

Aligning indicators across 39 districts proved to be a large undertaking. Initially, there were vast differences in the definitions of indicators, numerators, denominators, data sources, data collection methods, and reporting frequency. Tracking of definitional alignment was facilitated by a process in which organizations were invited to submit numerator and denominator counts summarized by month, for all months for which new data had been collected since the last data submission – including for metrics that were ‘close’ but not exactly definitionally aligned. Any deviations from the agreed-upon definitions were listed in the submission file. This allowed for the identification of opportunities for further alignment. An example of how an organization’s data collection processes changed as a result of the collaborative process is presented in the text box.

**Table:** 

**Text Box**: One organization’s experience with definitional alignment Lwala, based in Rongo, Kenya, took the following actions as a direct result of the Community Health Impact Data Collaborative: **Adjusted how existing data were reported to align with peers**, leading to new insight. Lwala measured the amount of weeks in a ‘first trimester’ differently for the Pregnancy Speed indicator. We adjusted our data timeframe to capture the same definition and in the process decided to start systematically tracking the first antenatal care (ANC) visit at 12, 14, and 16 weeks, as we found it better informed our program’s understanding of when a woman seeks her first ANC visit, a key focus area for Lwala programming. **Viewed data at a more granular level**, which offered fresh program trend and data quality opportunities. Coalition data were reported monthly with numerators and denominators. As Lwala generated this data, it promoted an ongoing review of granular trends and changes in the monthly data, between monitoring and evaluation and program teams. Moreover, understanding the data from a different angle also offered new vantage points for checking data quality; for example, if a denominator changes suddenly without explanation, a data quality assessment (DQA) exercise is initiated. **Redesigned parts of Lwala’s CHW digital data collection tool to optimize additional data for learning**. Lwala found we were not measuring certain indicators such as CHW speed of assessment. The Coalition exercise prompted Lwala to undertake a learning activity that included technical knowledge – that is, sharing meetings with another Coalition partner during which they demonstrate how they successfully collected this data in their digital system. As a result, Lwala is updating parts of CHW digital data collection tool to optimize measures of treatment speed and quality – and to further support CHWs who use the digital tool.

## Analysis

### Initial insights motivated subsequent analysis

Once the initial set of harmonized data was compiled, the relationship between two related indicators was examined: timeliness of pregnancy registration and the percentage of women giving birth in a health institution with skilled providers. The results of this analysis are forthcoming; however, it is already clear that opening the first group meeting with concrete analysis allowed us to; (i) generate momentum around what was possible with the data harmonization project and (ii) identify and overcome data reporting, cleaning, and aggregation challenges.

**Figure 3.**
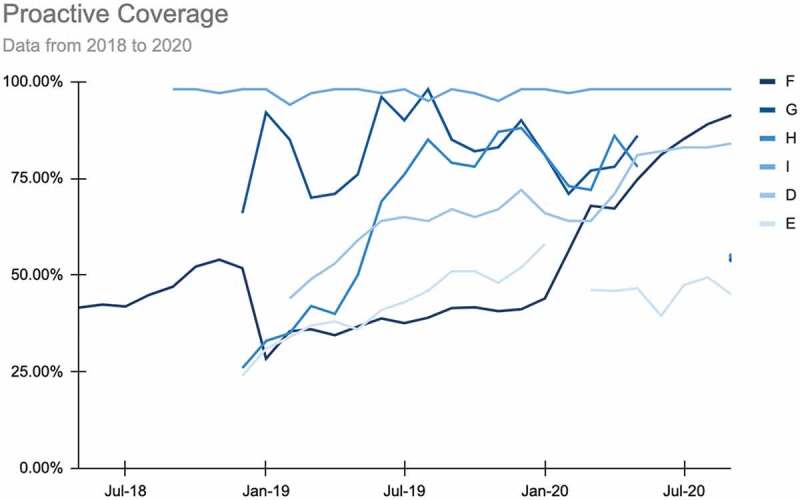
Proactive coverage data for 6 sites, 2018-2020.

An outlier analysis was likewise critical for generating momentum and hypotheses. The second data call focused on the trends in indicator number four and proactive coverage (Figure 3). The ‘high performer’ (organization F) and ‘big improver’ (organization I) presented on how they targeted improvements in that indicator, what unique elements of their model and/or context are likely barriers or facilitators to success, and how this might translate to other contexts. For example, one practice highlighted in this discussion was the use of a personalized performance feedback dashboard to increase home visits [15]. Together, the Collaborative used these presentations to share best practices and identify open questions and testable hypotheses.

These initial analyses helped identify and create momentum to improve the challenges in data quality and reporting, for example:

- To allow for confidence and speed in pooling, data require (i) cleaning and quality assurance at the organizational level and (ii) alignment in collection methods and periods (i.e. monthly vs. quarterly).
- To allow for a broader range of analyses, (i) raw numerators and denominators, rather than pre-calculated metrics, need to be shared; (ii) CHW counts provided; and (iii) data geographically disaggregated to allow for the observation of trends across different implementation sites for the same partner.

The identification and remediation of these challenges not only illustrate the value of the Collaborative for knowledge production, but also helps identify areas for data system strengthening at the organizational level.

### First published results

In the context of the COVID-19 pandemic, data harmonization and pooling of groundwork allowed for quick assessment of the pandemic’s impact across geographic regions.

**Figure 4.**
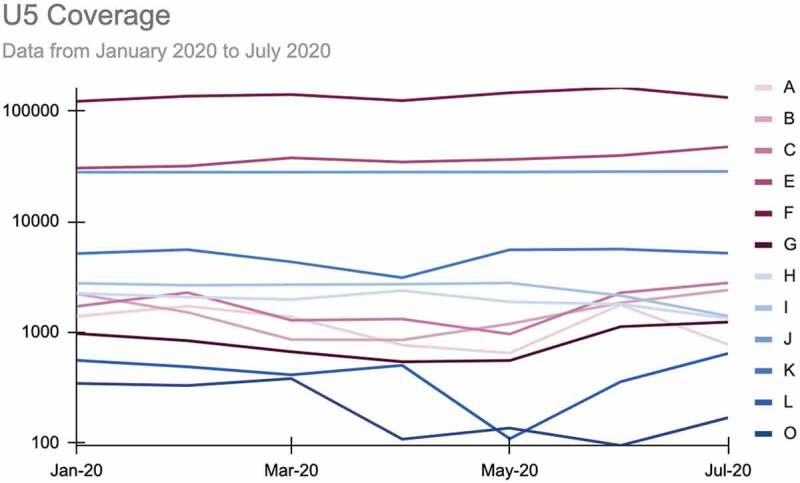
U5 coverage data for 12 sites, January-July 2020.

While preliminary studies modeled estimates quantifying disruptions to care using data collected at the facility level (DHIS2) or modeled estimates using survey data, no observational data or estimates looking at disruptions to care at the community level were initially available. Given that the majority of essential health services in many low- and middle-income countries were provided in the community before the onset of the pandemic [16], the Collaborative used its time series data to examine possible disruptions to the continuity of care at the community level (Figure 4).

The availability of a pre-existing multi-country data series allowed for the rapid generation and publication of real-time insights during a crisis. The results of the analysis [17] both underscore the avoidable nature of disruptions to care and, more broadly, illustrate the value of data on care provided by CHWs in better understanding the performance of the entire health system, particularly door-to-door care within communities.

## Discussion: the collaborative today

### Geographic scope

Since the data harmonization collaboration began, the Coalition has grown to more than 26 members, several of whom are in the process of being onboarded into the data harmonization Collaborative. Currently, the Collaborative includes data from nine partners, representing more than 8,300 CHWs in 39 districts accross eight countries (Togo, Kenya, Uganda, Malawi, Mali, Guatemala, Nepal, and Pakistan) (Table 2).

One notable aspect of the Collaborative is that it has meaningfully brought peer organizations together in a sector that is frequently characterized by competition and mistrust. While voluntary data-sharing would typically be seen as a risk in a highly competitive environment, this Collaborative has demonstrated that, with the right facilitation, the risk of data-sharing can be outweighed by the value of cross-site learning and the creation of new knowledge products that would be impossible for any given organization to release on its own.

### Next steps

The Coalition is committed to engage critically with the power dynamics within global health initiatives such as this one, and to ensure its practices combat, rather than perpetuate, systems and histories of exploitation.

During the first months of the Collaborative, partner organizations were represented by their research, monitoring and evaluation, learning, and/or data teams. These team members set the Collaborative’s priorities and were invited to participate in meetings and publications. The Collaborative quickly recognized, however, the need for a more intentional and equitable approach to engaging CHWs and other programmatic colleagues in this collaborative work. CHWs provide the services and acquire community-level programmatic data that make the data harmonization Collaborative possible, a contribution often undervalued by the norms and regulations in global health research. CHW supervisors and program managers ensure that coverage, speed, and quality care are provided to communities, and that the data collected are complete and reliable. In future, the Collaborative commits to more proactive creation of opportunities for CHWs, their supervisors, and/or program managers to participate directly in the Collaborative, its processes, and outputs.

This commitment has entailed engaging with questions of power across each stage of the Coalition’s collaboration and a shared agreement with the following changes in the planning, analysis, writing, and dissemination process.

### Planning and analysis

The Collaborative commits to ensuring that CHWs, their supervisors, and/or program managers are able to participate before, during, and after the Collaborative’s quality improvement sessions, in which data are interpreted and hypotheses for future research are generated.

In addition to ensuring real-time linguistic translation as needed, the Coalition will facilitate the interrogation of quantitative data and the interpretation of results by employing visual participatory analysis methods. These methods aim to engage CHWs as well as community members in the review and interpretation of data, providing collective opportunities to understand whether data correspond to the everyday experiences of individuals and communities [18]. The Collaborative will also extend the data harmonization initiative to include qualitative data sources, such as success stories from the frontlines of service delivery, interviews, and other forms of narrative accompaniment, which may help to better ‘make sense’ of organizational and pooled data from multiple perspectives and positionalities.

### Writing and dissemination

Where the Collaborative endeavors to publish, it will commit to inviting authorship contributions during the paper-writing phase in many languages and in non-written forms to ensure that diverse voices and perspectives are reflected in its outputs. The Collaborative also recommits to ensuring scientific outputs are shared deliberately with national government partners in the countries from which these data are derived, via dissemination workshops or meetings, as well as with communities themselves.

## Conclusions

This first foray into pooling data across members of the Community Health Impact Coalition produced promising results for quality improvement and generated a number of ideas for future forms of data engagement within the Collaborative. These pooled data will enable new opportunities for cross-site learning and contribute to an aggregate view of what can be achieved with high-impact community health systems worldwide. The Coalition’s commitment to equitable, intentional, and participatory knowledge co-production will continue to grow and evolve as the project progresses. The Coalition invites others to join us in expanding and refining the harmonization of service delivery indicators to improve the well-being of CHWs who deliver care with and for communities worldwide.

## Paper context

The WHO Guideline on CHWs revealed knowledge gaps about which health system practices promote quality care. Community Health Impact Coalition organizations deliver care in over 40 countries, representing an important learning opportunity. To leverage and learn from routine data, however, site-specific data must be harmonized and pooled. This article outlines (i) the rationale and methods used to establish a data harmonization and pooling Collaborative, (ii) early findings, (iii) lessons learned, and (iv) directions for future research.
